# Chinese Classical Formula Sijunzi Decoction and Chronic Atrophic Gastritis: Evidence for Treatment Approach?

**DOI:** 10.1155/2017/9012929

**Published:** 2017-08-24

**Authors:** Danan Gan, Aili Xu, Hongbo Du, Yong'an Ye

**Affiliations:** ^1^Department of Gastroenterology, Dongzhimen Hospital, Beijing University of Chinese Medicine, No. 5 Haiyuncang Street, Dongcheng District, Beijing 100700, China; ^2^Department of Gastroenterology, Wangjing Hospital, China Academy of Chinese Medical Sciences, Huajiadi Street, Chaoyang District, Beijing 100102, China

## Abstract

**Objective:**

This aim is to evaluate the effect of Sijunzi decoction (SJZD) treating chronic atrophic gastritis (CAG).

**Methods:**

We performed searches in seven databases. The randomized controlled trials (RCTs) comparing SJZD with standard medical care or inactive intervention for CAG were enrolled. Combined therapy of SJZD plus conventional therapies compared with conventional therapies alone was also retrieved. The primary outcome included the incidence of gastric cancer and the improvement of atrophy, intestinal metaplasia, and dysplasia based on the gastroscopy and pathology. The secondary outcomes were* Helicobacter pylori* clearance rate, quality of life, and adverse event/adverse drug reaction.

**Results:**

Six RCTs met the inclusion criteria. The research quality was low in the trials. For the overall effect rate, pooled analysis from 4 trials showed that modified SJZD plus conventional medications exhibited a significant improvement (OR = 4.86; 95% CI: 2.80 to 8.44;* P* < 0.00001) and without significant heterogeneity compared with the conventional medications alone. None reported the adverse effect.

**Conclusions:**

Modified SJZD combined with conventional western medicines appears to have benefits for CAG. Due to the limited number and methodological flaw, the beneficial and harmful effects of SJZD for CAG could not be identified. More high-quality clinical trials are needed to confirm the results.

## 1. Introduction

Chronic atrophic gastritis (CAG) is a common inflammatory condition typically characterized by the loss of gastric glandular structures or by glandular structures metaplastic atrophy [1]. The clinical symptoms include epigastric pain, fullness, belching, anorexia, and other nonspecific symptoms [2, 3]. Furthermore, it is worth noting that* helicobacter pylori* (HP) infection has a remarkable influence on the incidence of CAG. A systematic review published in 2010 reported that the rate ratio between HP infection and CAG incidence ranged from 2.4 to 7.6 [4]. In certain instances, a small subset of CAG cases eventually progress to gastric neoplasia [5, 6]. And the severity of CAG has been demonstrated to be a key risk factor for the development of gastric cancer from a 10-year prospective cohort study in Japan [7]. Therefore, the proper management of CAG will contribute to the prevention of gastric cancer.

In the viable therapies, pharmacotherapy is still dominant and has been widely applied in the treatment of CAG. These medications are regularly used to alleviate the clinical symptoms and improve quality of life, including acid-inhibitory drugs, HP eradication therapy, mucosal-protective agents, gastrointestinal prokinetic drugs, and digestants [8–11]. Additionally, antidepressant or antianxiety agent may be necessary for the CAG patients with obvious tendency of nervousness and emotional instability [12]. Mucosal-protective agents and proton pump inhibitor were most commonly used medications for chronic gastritis in China [13]. However, the medications still cannot meet clinical needs with respect to efficacy [14, 15]. And patients with long-term western medicine use such as proton pump inhibitor may have a higher possibility of experiencing either diffuse or linear/micronodular enterochromaffin-like cell hyperplasia [16]. In this situation, an increasing number of clinicians and patients are starting to choose herbal treatment for gastric inflammatory condition [17, 18]. As one of the most popular forms of alternative medicine, Chinese classical formula and materia medica have been gradually adopted in different cultures and regions [19, 20].

Sijunzi decoction (SJZD), a traditional Chinese herbal formula, has been frequently used for the treatment of various gastrointestinal disorders [21]. SJZD is composed of four commonly used herbs, including* Radix Ginseng* (Renshen),* Poria cocos* (Fuling),* Rhizoma Atractylodis Macrocephalae* (Baizhu), and* Radix Glycyrrhizae* (Gancao). According to the theory for Chinese prescription efficiency, SJZD is the representative formula for strengthening the spleen and replenishing Qi [22]. The existing researches on action mechanism have demonstrated that SJZD can ameliorate inflammation, reduce the histopathological injuries, enhance humoral and cellular immune responses, and improve immunological function of the rat through adjusting the genetic expression of JAK-STAT signal pathway [22–24].

In the last two decades, more and more clinical studies have reported the application of SJZD or modified SJZD for better effectiveness in patients with chronic gastritis or CAG, especially in China [25, 26]. Nevertheless, the evidence from the systematic review on SJZD for CAG is insufficient. To address these issues, this systematic review aims to synthesize available data and evaluate clinical evidence of SJZD treating CAG from randomized controlled trials (RCTs).

## 2. Methods

This study was designed and reported according to Preferred Reporting Items for Systematic Reviews and Meta-Analyses (PRISMA) statement recommendations [27].

### 2.1. Information Sources and Search Strategies

We performed searches in PubMed, EMBASE, Cochrane Library, Chinese National Knowledge Infrastructure (CNKI), Chinese Scientific Journal Database (VIP), Wanfang database, and Chinese Biomedicine Literature Database (SinoMed) from their inception through December, 2016. No restrictions were placed on age, gender, or duration of symptoms. But the search language was limited to English and Chinese. Searching strategies were made through the way of title/abstract, key words, and MeSH terms. The search terms “chronic gastritis”, “chronic atrophic gastritis”, “precancerous lesions of gastric cancer”,“atrophic”, “Sijunzi decoction”, “Sijunzi formula”, “Sijunzi tang”, “Sijunzi pill”, “Sijunzi powder”, “Sijunzi capsule”, “Sijunzi granule” and “random” were applied in various combinations to identify relevant literature. The titles and abstracts of the previous studies were retrieved using the reference management software NoteExpress version 2.0.

### 2.2. Inclusion Criteria and Exclusion Criteria

To be included in the systematic review, the studies had to meet the following criteria: (1) the type of design was RCT; (2) the articles were published in English or Chinese peer-reviewed journals; (3) the trials compared SJZD with standard medical care or inactive intervention(s) for CAG, such as triple therapy or placebo, and combined therapy of SJZD plus conventional therapies compared with conventional therapies alone was also retrieved; (4) outcome measurement used a validated tool. The primary outcome measures included the incidence of gastric cancer and the improvement of atrophy, intestinal metaplasia, and dysplasia based on the gastroscopy and pathology [15]. Subsequently, we could calculate the overall effect rate according to the improvement of gastroscopy and pathology. Histologic grading score mainly referred to the updated Sydney system [28]. The secondary outcomes were Hp clearance rate, quality of life, and adverse event/adverse drug reaction. In addition, the doctors might use SJZD directly or use modified SJZD (modify some Chinese herbs in SJZD) through judging the patients' clinical symptoms or signs in clinical practice. So the modified SJZD was also included in the review.

The exclusion criteria were listed as follows: (1) non-RCTs or quasi-RCTs and animal study; (2) journal or conference proceedings with no associated full-text article; (3) inappropriate intervention or control, such as SJZD combined with other alternative therapies (herbal formula, acupuncture, moxibustion, cupping, Taichi, Baduanjin, Wuqinxi exercise, etc.) which lacked evidence; (4) nonrecognized outcomes, for instance, self-compiled assessment scale which was not validated. Two authors (D. N. Gan and A. L. Xu) independently searched and selected the eligible trials according to the inclusion and exclusion criteria. Disagreement was resolved by discussion.

### 2.3. Data Extraction and Quality Assessment

Two authors (A. L. Xu and H. B. Du) extracted the data using a predetermined form. After extraction, data were compared by A. L. Xu, with disagreements being solved by consensus. We contacted the authors of the original articles when we needed to clarify the study data.

All the included studies were evaluated by using the criteria from the Cochrane Handbook for Systematic Review of Interventions [29]. The items reported random generation, allocation concealment, blinding of participants and personnel, blinding of outcome assessors, incomplete outcome data, selective reporting, and other bias. The evaluated domains were judged as low, high, or uncertain risk of bias. Where the two reviewers were uncertain or cannot agree on the quality of individual studies, a third reviewer (Y. A. Ye) would act as an arbiter.

### 2.4. Data Synthesis

All analyses were performed with the Review Manager 5.2.0 software (Cochrane Collaboration). We chose odds ratio (OR) to present dichotomous outcomes and mean difference (MD) to calculate continuous outcomes with 95% confidence interval (CI). The *χ*^2^ test and *I*^2^ scores were used to measure statistical heterogeneity. If the result was *P* < 0.1 and *I*^2^ ≥ 50%, the heterogeneity was considered to be high. Random or fixed effect model for meta-analysis of included trials was used based on the heterogeneity between their results. To decrease heterogeneity and increase reliability, subgroup analysis was performed for the comparable group.

## 3. Results

### 3.1. Description of the Included Trials

**Figure 1:**
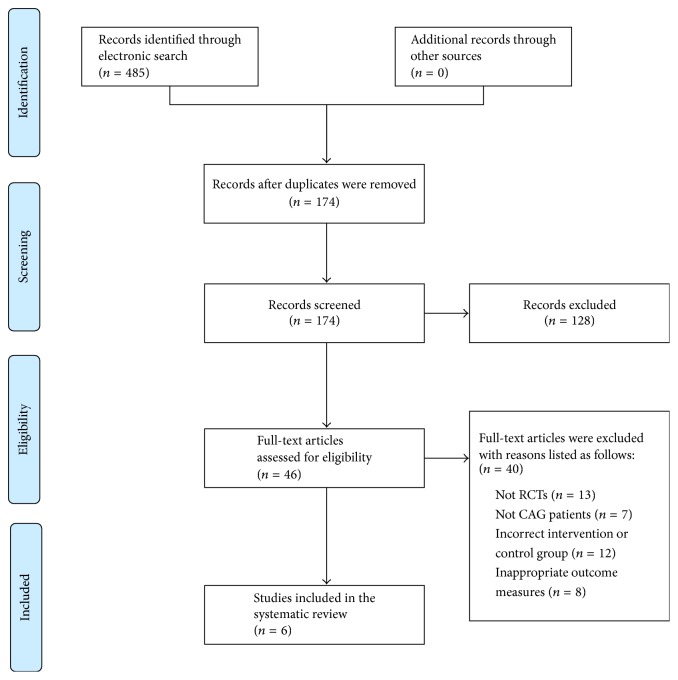
PRISMA 2009 flow diagram.

The details about the multistep literature screening process were outlined in Figure 1. We identified 485 new articles. Through removing the duplicated articles, 174 reports were reserved. After screening of titles and abstracts, we excluded 128 reports. Then, the remaining 46 were studied in detail and a further 40 were subsequently excluded. The reasons for exclusion were as follows: not RCTs (*n* = 13), not CAG patients (*n* = 7), incorrect intervention or control group (*n* = 12), and inappropriate outcome measures (*n* = 8). Eventually, 6 randomized trials that had been conducted in China and published in Chinese met our inclusion criteria [30–35]. They were published between 2009 and 2016. Of these 6 new trials, no trials were placebo-controlled.

### 3.2. Essential Characteristics of the Included Trials

Characteristics of the RCTs in this review were described in Table 1. The sample size ranged from 64 to 126 with a total size of 502. Three trials applied the diagnosis criterion from clinical research guideline on new drugs of traditional Chinese medicine [30–32], one trial used diagnosis criterion of Chinese digestive endoscopy association (gastroscopy diagnosis) and second national consensus meeting on chronic gastritis in China (pathology diagnosis) [34], one trial only mentioned pathological examination with the help of gastroscopy [35], and the other one did not report any criterion [33]. Five trials compared modified SJZD plus conventional medicines with conventional medicines alone [30, 32–35], and one trial compared modified SJZD with conventional medicines [31]. We summarized the composition of the formula in Table 2. In the control group, the medications were recommended by international or Chinese clinical practice guidelines.

The course of treatment varied from 3 to 24 weeks, but one trial did not report the treatment duration [33]. Five trials evaluated the overall effect rate including manifestations of gastroscopy and pathology [30–33, 35], and one trial assessed the histologic grading score [34], while the secondary outcome only observed Hp clearance rate in one trial [31].

### 3.3. Risk of Bias in the Included Trials

The methodological quality for the six included studies was presented in Table 3. The reporting quality was classified as high risk of bias in all the trials. The major reason for low quality was a lack of randomization and blinding. Only one trial reported the method generating random sequence [35], while the others simply mentioned that patients were randomly allocated without specific random method. The six trials were not explicit about the reporting of an appropriate method of allocation concealment, blinding of outcome assessor, and selective reporting. We considered the three items to be unclear risk of bias because of insufficient information. In the enrolled trials, no RCTs registered the research protocols. The item “blinding of participants and personnel” was judged as high risk of bias, because no placebo-controlled trials were designed and found. Only one trial described the dropout or withdrawal data in the article [30]. None of the trials had a pretrial sample size calculation. For the item “other bias,” only one trial did not report that the two groups had similarity at the baseline [33].

### 3.4. Efficacy of the Interventions

As for the existing different interventions, this study formed two separate comparisons: modified SJZD compared to conventional medicines and modified SJZD plus conventional medicines compared to conventional medicines alone.

#### 3.4.1. Modified SJZD Compared to Conventional Medicines

The meta-analysis was not designed between the two groups. Overall effect rate and Hp clearance rate were evaluated, respectively, in a trial [31]. After treatment for 4 weeks, modified SJZD monotherapy showed better effect on improving overall effect rate and Hp clearance rate compared to combination of the conventional drugs (domperidone, colloidal bismuth pectin, and omeprazole).

#### 3.4.2. Modified SJZD Plus Conventional Medicines Compared to Conventional Medicines Alone

The other 5 trials compared the effect of modified SJZD plus conventional medicines with conventional medicines alone [30, 32–35]. Histologic grading score was assessed in a trial [34]. The results indicated that modified SJZD plus conventional medications could improve significantly the histologic scores of atrophy, intestinal metaplasia, and dysplasia compared with the conventional medications (omeprazole, clarithromycin, and amoxicillin treatment for 14 days and folate treatment for 12 weeks) alone (*P* < 0.05).

**Figure 2:**
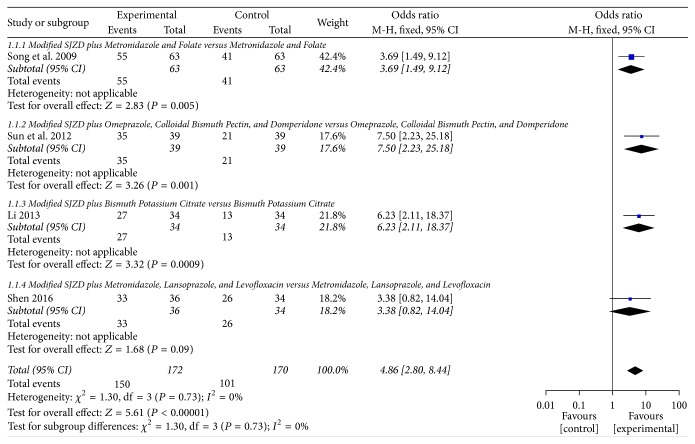
Forest plot of modified SJZD plus conventional medicines compared to conventional medicines* alone*; outcome: overall effect rate.

Overall effect rate was observed in the remaining 4 trials [30, 32, 33, 35]. Pooled analysis from 4 trials showed that modified SJZD plus conventional medications exhibited a significant improvement (OR = 4.86; 95% CI: 2.80 to 8.44; *Z* = 5.61, *P* < 0.00001) and without significant heterogeneity (*χ*^2^ = 1.30, *P* = 0.73; *I*^2^ = 0%) compared with the conventional medications. Fixed effect model was used to estimate the pooled effect. See Figure 2. A statistically significant difference between the intervention and control groups was also found in 3 trials [30, 32, 33].

(*1) Modified SJZD plus Metronidazole and Folate versus Metronidazole and Folate*. After treatment for 24 weeks, there was statistically significant difference between the combination-therapy group and western medicines alone (OR = 3.69; 95% CI: 1.49 to 9.12; *Z* = 2.83, *P* = 0.005) [30].

(*2) Modified SJZD plus Omeprazole, Colloidal Bismuth Pectin, and Domperidone versus Omeprazole, Colloidal Bismuth Pectin, and Domperidone*. After treatment for 3 weeks, there was statistically significant difference between the combination-therapy group and conventional medicines alone on overall effect rate (OR = 7.50; 95% CI: 2.23 to 25.18; *Z* = 3.26, *P* = 0.001) [32].

(*3) Modified SJZD plus Bismuth Potassium Citrate versus Bismuth Potassium Citrate*. Modified SJZD plus bismuth potassium citrate was better than bismuth potassium citrate in improving the clinical overall effect rate (OR = 6.23; 95% CI: 2.11 to 18.37; *Z* = 3.32,* P *= 0.0009) [33].

(*4) Modified SJZD plus Metronidazole, Lansoprazole, and Levofloxacin versus Metronidazole, Lansoprazole, and Levofloxacin*. After treatment for 12 weeks, there was no statistical significance between the two groups (OR = 3.38; 95% CI: 0.82 to 14.04; *Z* = 1.68, *P* = 0.09) [35].

Additionally, we did not find any assessment on the incidence of gastric cancer and quality of life in these identified studies.

### 3.5. Adverse Effect of the Interventions

None of the trials reported the adverse event or adverse drug reaction in the previous studies.

## 4. Discussion

### 4.1. Overview of Findings

In the systematic review, we included 6 RCTs following the inclusion criteria. All the trials used the modified SJZD as the main intervention. Only one trial compared modified SJZD with conventional medicines, including domperidone, colloidal bismuth pectin, and omeprazole [31]. The results showed that modified SJZD was more effective than conventional medicines in improving overall effect rate and Hp clearance rate. Nevertheless, the analytical data was extracted from one trial with a small sample size, and the trial did not perform blinding. Five trials compared the clinical efficacy of modified SJZD plus conventional medicines with conventional medicines alone [30, 32–35]. One trial found that modified SJZD plus conventional medications could improve significantly the scores of histopathology compared with the conventional medications, including HP eradication therapy and folate treatment [34]. But the methodological flaw such as randomization and blinding was also found in the trial. The meta-analysis indicated that modified SJZD plus conventional medications had a significant improvement compared with the conventional medications in improving overall effect rate [30, 32, 33, 35]. Although the pooled analysis created a positive result, it was still difficult to draw a definite conclusion because of the limited sample size of outcome events (150 versus 101) and low-quality studies.

Meanwhile, no extra information on the incidence of gastric cancer, quality of life, and adverse events/adverse drug reaction could be available to assess the efficacy or adverse effect of SJZD for CAG. The course of treatment was also inconsistent in the included trials and might affect the effectiveness of Chinese herbal formula.

### 4.2. Comparison with the Previous Systematic Review

So far there was a systematic review reporting the modified SJZD treating CAG that preceded our study [36]. Both the two systematic reviews and meta-analysis demonstrated that modified SJZD plus conventional western medicine can significantly improve the overall effect in treating patients with CAG compared with conventional western medicine.

However, the differences could be distinguished between the two reviews. Firstly, the previous review included 7 trials, while 3 trials in that review were enrolled in our study [31–33]. Four trials were excluded, because 2 trials chose unconventional treatment as control group, while the others did not design recognized outcome measures. Secondly, we searched the literatures from their inception until December, 2016. One article published in 2009 [30] and two articles published in 2016 [34, 35] were screened. Thirdly, the outcomes including Hp clearance rate and histologic grading score were reported in our study.

### 4.3. Limitations and Implications

The quality of each of the included trials was evaluated by using the Cochrane Collaboration's tool. The methodology evaluation showed a high risk of bias in domain of blinding for participants and personnel, which directly weakened the strength of the positive results. In spite of difficulties, the double-blinding clinical trial should be strongly recommended to confirm the absolute effect of Chinese herbal formula [37].

SJZD came from the Chinese pharmacopoeia named “*Tai Ping Hui Min He Ji Ju Fang*” in Song dynasty. For SJZD, two limitations or questions should be noted to this review.* Radix Ginseng* (Renshen) was substituted by* Radix Codonopsis* (Dangshen) in the four studies [30, 32, 34, 35]. According to the traditional Chinese medicine theory, the role of strengthening the spleen and replenishing Qi might be weakened. On the other hand, the physicians always modified some herbs based on the original prescription of SJZD including just four herbs. In our studies, the number of modified herbs ranged from 3 to 10 kinds. Therefore, the modified SJZD was difficult to be standardized and the clinical effect of the interventions should be different from each other.

Additionally, we only searched electronic databases but did not conduct a manual retrieval, which might leave out the relevant clinical trials. As the sample size of the included studies was relatively small, we were unable to determine the effect estimates of the intervention.

Based on the existing problems of the current studies, more and more rigorous RCTs including multicenter, placebo-controlled clinical trials are needed to be launched to produce higher quality evidence. The study protocol of traditional Chinese medicine clinical trials should be registered or published in the future [38]. The appropriate randomization method and sample size calculation would be applied. In respect of trial reporting, the researchers should follow the checklist of the Consolidated Standards for Reporting Trials (CONSORT) [39].

### 4.4. Conclusion

Modified SJZD combined with conventional western medicines appears to have benefits for the patients with CAG compared with conventional western medicines. Due to the limited number and methodological flaw of the previous studies, the beneficial and harmful effects of SJZD or modified SJZD for CAG could not be identified. More rigorous RCTs and further clinical evidence are needed to confirm the results.

## Figures and Tables

**Table 1:** Characteristics of the included studies.

Study ID	Sample size (*T*/*C*)	Diagnosis criteria	Intervention	Control	Course of treatment	Outcome assessment
Song et al., 2009 [30]	68 (34/34)	CRGNDTCM (including gastroscopy and pathology diagnosis)	Modified SJZD + control	Metronidazole (200 mg, twice a day), 1 week, and folate (10 mg, three times a day), 24 weeks	24 weeks	Overall effect rate

Hu, 2011 [31]	96 (48/48)	CRGNDTCM (including gastroscopy and pathology diagnosis)	Modified SJZD	Domperidone (10 mg, three times a day), colloidal bismuth pectin (200 mg, four times a day), and omeprazole (20 mg, twice a day)	4 weeks	Overall effect rate Hp clearance rate

Sun et al., 2012 [32]	70 (36/34)	CRGNDTCM (including gastroscopy and pathology diagnosis)	Modified SJZD + control	Omeprazole (30 mg, once a day) and colloidal bismuth pectin (120 mg, three times a day) Abdominal distension or regurgitation onset: domperidone (10 mg, three times a day) HP infection: amoxicillin (500 mg, three times a day) and tinidazole (1 g, three times a day)	3 weeks	Overall effect rate

Li, 2013 [33]	126 (63/63)	Not reported	Modified SJZD + control	Bismuth potassium citrate (300 mg, three or four times a day)	Not reported	Overall effect rate

Zhang, 2016 [34]	64 (32/32)	Gastroscopy diagnosis: DCBDACMA Pathology diagnosis: SNCMCG	Modified SJZD + control	Omeprazole (20 mg, twice a day), clarithromycin (0.5 g, once a day), amoxicillin (1 g, twice a day), 14 days; and folate (10 mg, three times a day), 12 weeks	12 weeks	Histologic grading score

Shen, 2016 [35]	78 (39/39)	Not reported, but mentioned pathological examination with the help of gastroscopy	Modified SJZD + control	Metronidazole (400 mg, twice a day), Lansoprazole (30 mg, twice a day), and Levofloxacin (200 mg, twice a day)	12 weeks	Overall effect rate

*T*: treatment group; *C*: control group; CRGNDTCM: clinical research guideline on new drugs of traditional Chinese medicine; DCBDACMA: diagnosis criterion of Chinese digestive endoscopy association; SNCMCG: second national consensus meeting on chronic gastritis in China; SJZD: Sijunzi decoction.

**Table 2:** Composition of formula.

Study ID	Formula	Composition of formula
Song et al., 2009 [30]	Modified SJZD	*Radix Codonopsis* (Dangshen) 9 g, *Poria Cocos* (Fuling) 9 g, *Rhizoma Atractylodis Macrocephalae* (Baizhu) 9 g, *Radix Glycyrrhizae* (Gancao) 6 g, *Trogopterus xanthipes *Milne-Edwards (Wulingzhi) 8 g, *Rhizoma Chuanxiong* (Chuanxiong) 8 g, *Herba Hedyotidis Diffusae* (Baihuasheshecao) 6 g

Hu, 2011 [31]	Modified SJZD	*Radix Ginseng* (Renshen) 15 g, *Poria Cocos* (Fuling) 15 g, *Rhizoma Atractylodis Macrocephalae* (Baizhu) 15 g, *Radix Glycyrrhizae* (Gancao) 6 g, *Radix Astragali* (Huangqi) 30 g, *Pericarpium Citri Reticulatae* (Chenpi) 12 g, *Radix Glehniae* (Beishashen) 12 g, *Radix Ophiopogonis* (Maidong) 12 g, *Herba Dendrobii* (Shihu) 12 g, *Fructus Amomi* (Sharen) 6 g, *Fructus Hordei Germinatus *(Maiya) 30 g, *Fructus Setariae Germinatus* (Guya) 30 g

Sun et al., 2012 [32]	Modified SJZD	*Radix Codonopsis* (Dangshen) 20 g, *Poria Cocos* (Fuling) 20 g, *Rhizoma Atractylodis Macrocephalae* (Baizhu) 15 g, *Radix Glycyrrhizae* (Gancao) 5 g, *Fructus Aurantii Immaturus* (Zhishi) 15 g, *Radix Salviae Miltiorrhizae* (Danshen) 10 g, *Radix Paeoniae Rubra* (Chishao) 15 g, *Radix Bupleuri* (Chaihu) 5 g

Li, 2013 [33]	Modified SJZD	*Radix Ginseng* (Renshen) 10 g, *Poria Cocos* (Fuling) 10 g, *Rhizoma Atractylodis Macrocephalae* (Baizhu) 10 g, *Radix Glycyrrhizae* (Gancao) 5 g, *Radix Astragali* (Huangqi) 10 g, *Radix Paeoniae Alba* (Baishao) 10 g, *Semen Lablab Album* (Baibiandou) 10 g

Zhang, 2016 [34]	Modified SJZD	*Radix Codonopsis* (Dangshen) 10 g, *Poria Cocos* (Fuling) 10 g, *Rhizoma Atractylodis Macrocephalae* (Baizhu) 10 g, *Radix Glycyrrhizae* (Gancao) 6 g, *Radix Astragali* (Huangqi) 10 g, *Rhizoma Curcuma* (Eshu) 10 g, *Rhizoma Chuanxiong* (Chuanxiong) 10 g, *Herba Hedyotidis Diffusae* (Baihuasheshecao) 10 g

Shen, 2016 [35]	Modified SJZD	*Radix Codonopsis* (Dangshen) 15 g, *Poria Cocos* (Fuling) 15 g, *Rhizoma Atractylodis Macrocephalae* (Baizhu) 15 g, *Radix Glycyrrhizae* (Gancao) 10 g, *Rhizoma Cyperi* (Xiangfu) 15 g, *Cortex Magnoliae Officinalis* (Houpu) 15 g, *Radix Angelicae Sinensis* (Danggui) 15 g, *Radix Paeoniae Alba* (Baishao) 15 g, *Rhizoma Zingiberis* (Ganjiang) 10 g, *Rhizoma Pinelliae* (Banxia) 10 g, *Fructus Aurantii* (Zhike) 10 g, *Rhizoma Coptidis* (Huanglian) 7 g, *Fructus Amomi* (Sharen) 6 g

**Table 3:** Risk of bias assessment based on the Cochrane handbook.

Included studies	Random sequence generation	Concealment of allocation	Blinding of participants and personnel	Blinding of outcome assessment	Incomplete outcome data	Selective reporting	Other bias	Risk of bias
Song et al., 2009 [30]	?	?	—	?	+	?	+	High
Hu, 2011 [31]	?	?	—	?	?	?	+	High
Sun et al., 2012 [32]	?	?	—	?	?	?	+	High
Li, 2013 [33]	?	?	—	?	?	?	?	High
Zhang, 2016 [34]	?	?	—	?	?	?	+	High
Shen, 2016 [35]	+	?	—	?	?	?	+	High

+: low risk of bias; —: high risk of bias; ?: unclear risk of bias.
